# Pan-cancer analysis of oncogenic role of Programmed Cell Death 2 Like (PDCD2L) and validation in colorectal cancer

**DOI:** 10.1186/s12935-022-02525-x

**Published:** 2022-02-25

**Authors:** Huabin Gao, Cheng Xu, Jiangtao Liang, Songhan Ge, Fenfen Zhang, Ying Tuo, Huijuan Shi, Anjia Han

**Affiliations:** Department of Pathology, The First Affiliated Hospital, Sun Yat-Sen University, 58, Zhongshan Road II, Guangzhou, 510080 China

**Keywords:** PDCD2L, Pan-cancer analysis, Oncogenic role, Colorectal cancer

## Abstract

**Background:**

Programmed Cell Death 2 Like (PDCD2L) correlates with cell proliferation, apoptosis and mouse embryonic development. However, the role of PDCD2L in human cancers is unclear.

**Methods:**

Multiple bioinformatic methods, in vitro function experiments and validation were performed to clarify the oncogenic role of PDCD2L in human cancers.

**Results:**

Our study found that PDCD2L was aberrantly expressed in multiple types of human cancers, and associated with clinical stage and molecular subtype. Furthermore, overexpression of PDCD2L predicted poor overall survival in adrenocortical carcinoma(ACC), kidney chromophobe(KICH), acute myeloid leukemia(LAML), brain lower grade glioma(LGG),liver hepatocellular carcinoma(LIHC), mesothelioma(MESO), uveal melanoma(UVM) and poor diseases free survival in ACC, bladder urothelial carcinoma(BLCA), cervical squamous cell carcinoma and endocervical adenocarcinoma (CESC), kidney renal clear cell carcinoma(KIRC), kidney renal papillary cell carcinoma(KIRP), LGG, LIHC, and UVM. PDCD2L expression was negatively associated with cancer associated fibroblast in breast invasive carcinoma (BRCA), lung squamous cell carcinoma (LUSC), sarcoma (SARC), stomach adenocarcinoma (STAD) and testicular germ cell tumors (TGCT). Mechanically, we found that PDCD2L expression was associated with apoptosis, invasion and cell cycle by investigating single cell sequencing data. For further validation, PDCD2Lwas highly expressed in colorectal cancer (CRC) cell lines and tissue samples compared with the normal colon cell line and non-tumor adjacent colorectal mucosa tissues. PDCD2L knockdown induced the apoptosis and proliferation of CRC cells.

**Conclusions:**

Our study shows that the oncogenic role of PDCD2L in various cancers and PDCD2L could be served as a biomarker of CRC.

**Supplementary Information:**

The online version contains supplementary material available at 10.1186/s12935-022-02525-x.

## Introduction

Cancer incidence is growing rapidly worldwide, female breast cancer has the highest incidence (11.7%), followed by lung cancer (11.4%) and colorectal cancer (10.0%). As a major cause of death, cancers kill almost 10.0 million people in 2020.The top 3 tumor-related mortality rates are lung cancer(18%), colorectal cancer(9.4%), and liver cancer(8.3%) [1]. Accordingly, given the incidence and mortality of cancers, it is urgent to find perfect biomarkers for making diagnosis and predicting the prognosis in various cancers.

Programmed Cell Death 2 Like (PDCD2L) is a coding protein, which also has alias symbols MGC13096. PDCD2Lcontaining PDCD2(C) domain and the expression of PDCD2L in HEK293T cells did not change significantly when apoptosis was induced by different treatments [2]. Augmented levels of MGC13096 protein inhibit proliferation of HEK293T cells, while up-regulation of MGC13096 block cell cycle progression at S phase [3]. Intriguingly, Ye et al. showed that up-regulation of PDCD2L in pancreatic beta-cell enhanced palmitate-induced apoptotic rate, and down-regulation of PDCD2L partially diminished the apoptosis induced by palmitate. This study revealed that PDCD2L might play an important role in apoptosis of pancreatic beta-cell [4]. Moreover, PDCD2L plays a key role during post-implantation embryonic development, which suggests that PDCD2L is evolutionarily conserved in development [5]. PDCD2L expression is related with cell proliferation, apoptosis, and mouse embryonic development. However, the role of PDCD2L is still unknown in various cancers.

Here, we explored the expression and prognostic value of PDCD2L in multiple cancers. Gene Ontology (GO) terms and Kyoto Encyclopedia of Genes and Genomes (KEGG)pathways were performed to explore the underlying molecular mechanisms of PDCD2L and its binding proteins. Then we explored the correlation between PDCD2L expression and immune infiltration. Moreover, single cell sequencing data was applied to investigate relevant cancer cell status of PDCD2L. We further verify PDCD2L expression in CRC cell lines and tissues by real-time quantitative PCR (RT-qPCR), western blot (WB), and immunohistochemistry (IHC) staining. We also analyzed the role of PDCD2L in CRC progression in vitro.

## Material and methods

### PDCD2L gene expression analysis based on pan-cancer database

PDCD2L expression data in pan-cancer tissues and corresponding adjacent tissues were downloaded from ONCOMINE website (https://www.oncomine.org/resource/login.html) [6, 7]and TIMER2.0 website (http://timer.cistrome.org/) [8–10].The threshold settings of ONCOMINE were followed: p-value of 0.001, fold change of 2.0,gene ranking of top 10%, and data type of all. Expression data of PDCD2L in different clinical stages of cancers were obtained from GEPIA2.0 website (http://gepia2.cancer-pku.cn/#index) [11].We also downloaded the expression data of PDCD2L in different molecular subtypes of tumor from TISDB website (http://cis.hku.hk/TISIDB/index.php) [12].TISDB is a website for the cross-linking analysis of tumor and immunity, which includes a variety of data, such as the results reported in PubMed database, high-throughput sequencing data, exon and RNA sequencing data of immunotherapy patients, genome, transcription group and clinical characteristics of patients downloaded from TCGA, and public databases.

### Survival and prognosis analysis

Overall survival data and diseases free survival data were downloaded from GEPIA2. The median expression of PDCD2L was divided into high PDCD2L expression group and low PDCD2L expression group. Log-rank test was used to analyze the relationship between PDCD2L expression and the survival rate of patients with different tumors.

### PDCD2L-related functional enrichment analysis

We analyzed the proteins binding to PDCD2L from STRING website (https://string-db.org/) and the version was 11.0 (archived version) [13].Then we set the following threshold to obtain the binding proteins: network type of full network, active interaction sources of experiments, minium required interaction score of low confidence (0.150).The meaning of network edges was set as evidence, also Max number of interactors to show was set as no more than 20 interactors. We found 18 proteins that bind to PDCD2L, which were validated by experiment. Furthermore, we analyzed the genes which have similar expression pattern with PDCD2L in pan-cancer by GEPIA2, and the top 100 genes were selected as candidate genes. GO enrichment analysis and KEGG pathway analysis of PDCD2L, 18 PDCD2L-binding proteins and 100 candidate genes (including the common gene LSM14A) were performed on DAVID website (https://david.ncifcrf.gov/) [14, 15].

### Immune infiltration analysis

TIMER2.0 provides a variety of immune deconvolution methods including TIMER, CIBERSORT, EPIC, MCPCOUNTER, XCELL, TIDE, etc., which provide approach for analyzing the immune, clinical and genetic characteristics of different tumors, comprehensively. The relationship between PDCD2L expression and immune infiltration of various cancers was analyzed by TIMER2.0 website, and the correlation between PDCD2L expression and cancer associated fibroblast (CAF) of various cancers was explored. Moreover, the association between PDCD2L expression and different tumor immune subtypes was analyzed by TISDB website. Different immune types are summarized as followed:C1 (wound healing), C2(IFN-gamma dominant), C3(inflammatory), C4(lymphocyte depleted), C5(immunologically), C6(TGF-b dominant).

### Single cell sequencing data analysis

CancerSEA (http://biocc.hrbmu.edu.cn/CancerSEA/home.jsp) is a specialized database for single cell sequencing, which can provide different functional status of cancer cells at the single cell level [16].We downloaded the correlation data between PDCD2L expression and different tumor functional status based on single cell sequencing data from CancerSEA and drew a heatmap. The t-SNE diagrams of all individual cells were obtained directly from the CancerSEA website.

### Clinical CRC tissue samples

Totally, 157 cases of paraffin embedding CRC specimens and 140 cases of non-tumor adjacent colorectal mucosa tissues (which included 126 paired CRC specimens) were obtained from our Department of Pathology, the first Affiliated Hospital, Sun Yat-Sen University, Guangzhou, China, between January 2013 and December 2013. Among them, 97 cases contain available follow-up information. The histopathology of CRC was determined by two experienced pathologists based on the criteria of the World Health Organization. We also obtained Prior patient consent and approval from the Institutional Research Ethics Committee.

### Cell lines and siRNA transfection

Colorectal cancer cell lines including HT29, HCT116, LOVO, RKO, SW480, SW620, and T84 were purchased from Cell Bank of Chinese Scientific Academy in 2017 and 2018 (Shanghai, China), colon mucosal epithelial cell line-NCM460 was purchased from INCELL Company (San Antonio, USA). The above cell lines were cultured in Dulbecco’smodified Eagle’s medium (DMEM) (Invitrogen, Carlsbad, CA) with 10% fetal bovine serum (Gibco, LifeTechnologies, CA) and 100 μg/ml penicillin–streptomycin. Cells were cultured at 37 °C with5% CO2 in a humidified incubator. siRNAs were purchased from RIBOBIO (Gunagzhou, China), then transfected with Lipofectamine3000reagent. The target sequence of PDCD2L siRNA were as followed: negative control:siN0000001-1–5, si-PDCD2L-1:GCTCAAGAGTGCTAATTTA, si-PDCD2L-2:GGACTATCAGCAGAGAGAA, and si-PDCD2L-3:GGAACAATTCTAGTTTACA.

### RNA extraction and quantitative RT-PCR

CRC cells or fresh tissues were collected and added with Trizol reagent according to the manufacturer’s guidelines. RNA concentration was measured by nanodrop one, and then reverse transcribed into cDNA. Then we performed quantitative RT-PCR by SYBR Premix ExTaq II (Takara, Osaka,Japan). GAPDH was used as normalization. The primers of quantitative RT-PCR are purchased from SANGON BIOTECH (Shanghai, China) and the sequences were as followed: PDCD2L-forward: GCGAATTGCTGCTTGTCAGG, PDCD2L-reverse: GAGCTCGGTGACTTCTGATGTAGG, GAPDH-forward: CAGGAGGCATTGCTGATGAT, GAPDH-reverse: GAAGGCTGGGGCTCATTT. All experiments were performed three times in triplicate.

### Western blot

Cells were collected from tissues using lysis buffer. Then we added 40 µg of protein per sample for SDS-PAGE separation and blot onto PVDF membranes. After blocking by 5% milk for 1 h at room temperature, membranes were incubated with primary antibodies PDCD2L(#A7394, Abclonal), P53(#10,442–1-AP, proteintech), c-Myc(#9402, Cell Signaling Technology), and GAPDH(#5174, Cell Signaling Technology) overnight at 4 °C. Then the membranes were incubated with HRP-linked secondary antibody (#7074, Cell Signaling Technology) at room temperature for 1 h. Finally, we obtained the signal from chemiluminescence detection reagent (Millipore).

### Immunohistochemistry staining and evaluation

Immunohistochemistry staining was performed as we previously described [17].The working concentration of PDCD2L(#A7394, Abclonal) was 1:200. The immunohistochemical score was obtained by the percentage score of positive tumor cells x intensity score. Among them, the percentage of positive tumor cells was scored from 0–1. The positive intensity was identified as 0 as negative, weak positive as 1, moderate positive as 2, and strong positive as 3. The immunohistochemical score was completed by two experienced pathologists.

### Apoptosis assay

The cells were digested by trypsin without EDTA, washed twice by phosphate buffered saline (PBS). Then we performed apoptosis assay using Annexin V-APC/PI Apoptosis Detection Kit (#KGA1030-50, KeyGen, Nanjing, China) according to the manufacturer’s guidelines, and detected on the flow cytometry. The experiments were performed three times in triplicate.

### Proliferation assays

CRC cell viability were performed by using Cell Counting Kit-8 (CCK-8) (#B34304, bimake). 1 × 10^3^/well of cells were collected and seeded into a 96 well plate. Then absorbance values were measured every day for 5 days according to manufacturer’s protocol. DNA synthesized rate of CRC cells (1 × 10^4^/well) was analyzed by 5-ethynyl-20-deoxyuridine (EdU) assay kit (#C10310, Ribobio, Guangzhou, China) following the manufacturer’s guidelines. Cell proliferation activity were assessed by the ratio of EDU positive cells (red fluorescence) to Hoechest-stained cells (blue fluorescence). The experiments were performed three times in triplicate.

### Statistical analysis

Unpaired t-tests were applied to compare the difference between two groups and data were presented as the mean ± standard deviation. Spearman’s rank correlation coefficient was applied to evaluate the correlation between two groups. The association between prognosis of patients and PDCD2Lexpression levels was assessed using Kaplan–Meier method. p < 0.05 was considered as a statistically significant difference.

## Results

### Aberrant expression of PDCD2L in pan-cancer

**Fig. 1:**
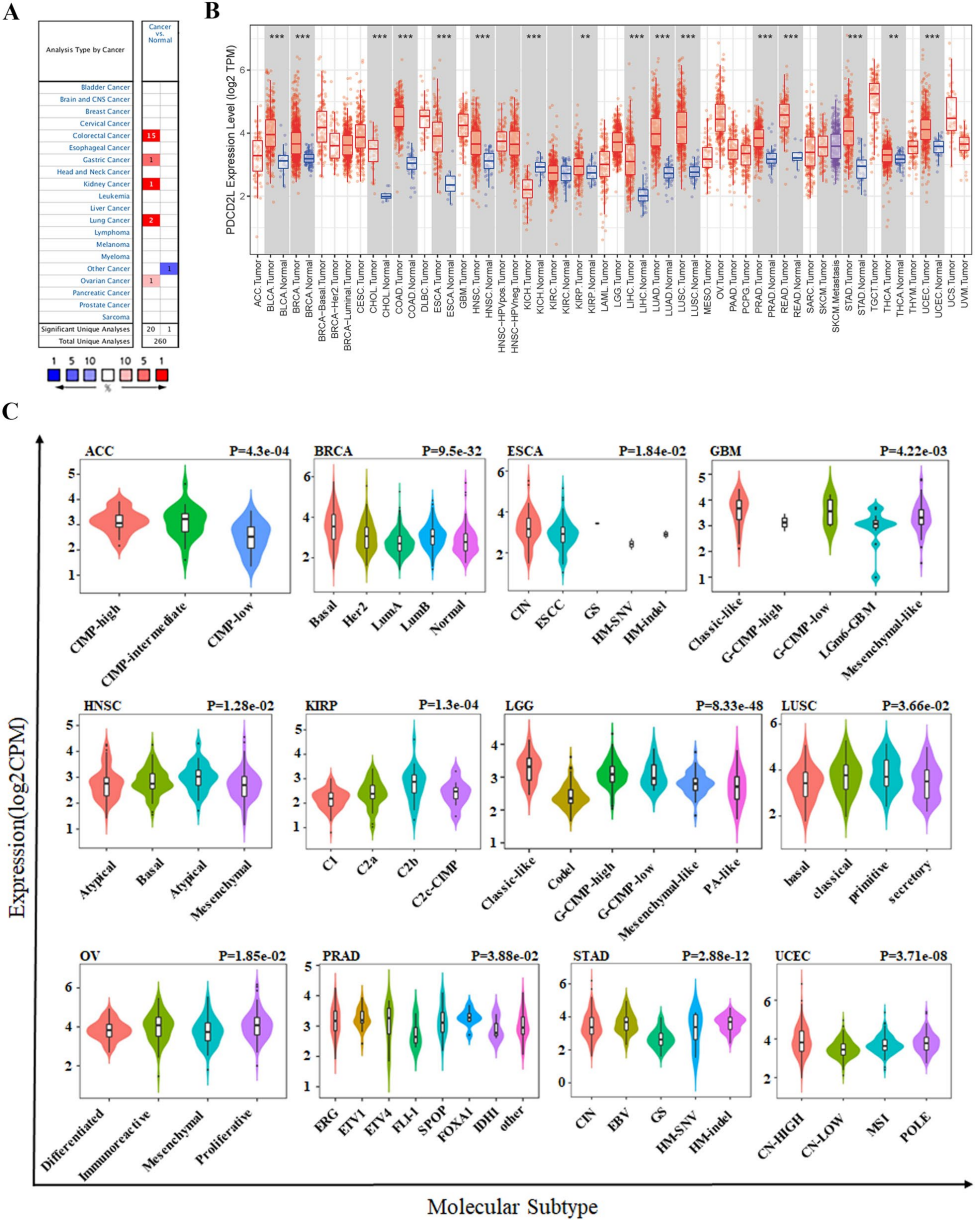
Aberrant expression of PDCD2L in pan-cancer. **A** PDCD2L expression in human cancers from ONCOMINE database. **B** PDCD2L expression in different cancers from TCGA based on TIMER2.0 (** P < .01; *** P < .001). **C** PDCD2L expression in clinic stage of ACC, KIRC, KIRP and LUSC according to GEPIA2. **D** PDCD2L expression in different molecular subtype of ACC, BRCA, ESCA, GBM, HNSC, KIRP, LGG, LUSC, OV, PRAD, STAD and UCEC by TISDB analysis

Firstly, we analyzed PDCD2L expression in pan-cancer through ONCOMINE website. The database provided gene expression of various cancers and corresponding normal samples. Results revealed thatPDCD2Lwas up-regulated in CRC, gastric cancer, kidney cancer and ovarian cancer while down-regulated in other cancers (Fig. 1A). Furthermore, we validated PDCD2L expression in pan-cancer by TIMER2.0. Consistent with our above finding, PDCD2L expression was increased in BLCA, BRCA, Cholangio carcinoma (CHOL), Colon adenocarcinoma(COAD), Esophageal carcinoma (ESCA), Head and Neck squamous cell carcinoma (HNSC), HNSC-HPVpos, KICH, KIRC, KIRP, LIHC, Lung adenocarcinoma (LUAD), LUSC, Prostate adenocarcinoma (PRAD), Rectum adenocarcinoma (READ), STAD, Thyroid carcinoma (THCA) and Uterine Corpus Endometrial Carcinoma (UCEC) (Fig. 1B).To explore the relationship between PDCD2L expression and clinical stage of pan-cancer, we performed further analysis by GEPIA2. The result suggested that PDCD2L expression was significantly associated with clinical stages of ACC, KIRC, KIRP and LUSC (Fig. 1C).While PDCD2L expression was not significantly associated with clinical stages of other cancers including BLCA, BRCA, CESC, CHOL, COAD, Lymphoid Neoplasm Diffuse Large B-cell Lymphoma (DLBC), ESCA, HNSC, KICH, LIHC, LUAD, Ovarian serous cystadenocarcinoma (OV),Pancreatic adenocarcinoma (PAAD), READ, Skin Cutaneous Melanoma (SKCM), STAD, TGCT, THCA, UCEC and Uterine Carcinosarcoma (UCS) (data not shown).We also performed another analysis through TISDB, which revealed that PDCD2L expression was significantly associated with the molecular subtype of ACC, BRCA, ESCA, Glioblastoma multiforme (GBM), HNSC, KIRP, LGG, LUSC, OV, PRAD, STAD and UCEC (Fig. 1D).While the expression of PDCD2L was not associated with the molecular subtype of COAD, LIHC, pheochromocytoma and paraganglioma(PCPG), READ and SKCM (Additional file 1: Figure S1).Taken together, compared with adjacent normal tissues, PDCD2L expression was up-regulated in various cancers and PDCD2Lmight be serve as an oncogene in these cancers.

### Prognostic analysis of PDCD2L expression in pan-cancer

**Fig. 2:**
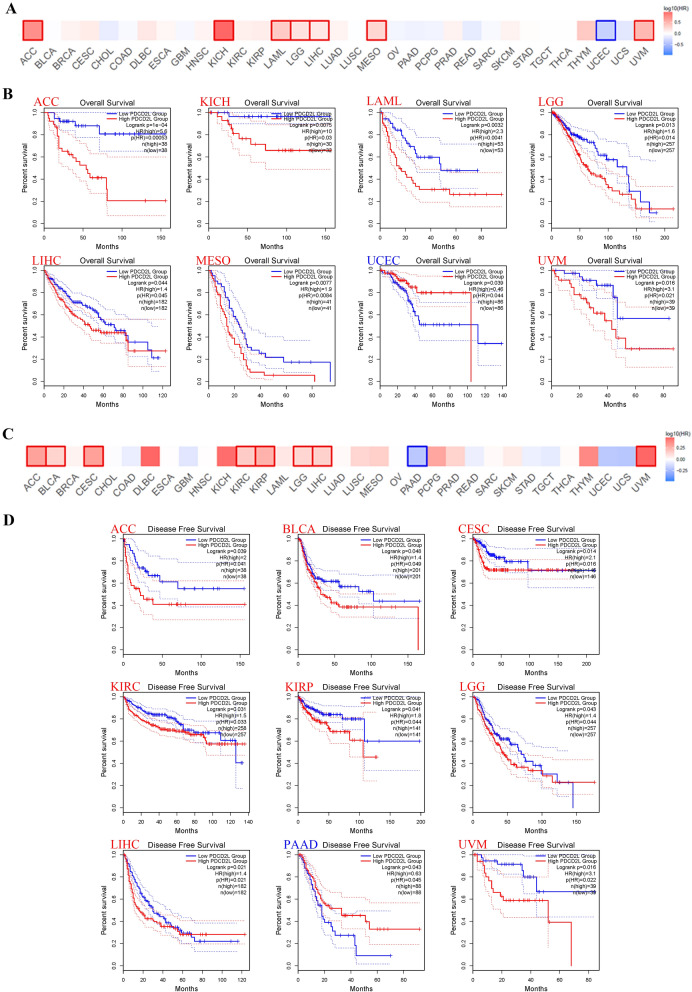
Prognostic value of PDCD2L expression in pan-cancer. Relationship between PDCD2L expression in different tumors from TCGA and overall survival (**A**, **B**) or diseases free survival (**C**, **D**) by GEPIA2. Only positive data of survival map and Kaplan–Meier curves were shown

To explore the relationship between PDCD2L expression and prognosis of patients in pan-cancer, we performed survival analysis by GEPIA2.The results suggested that high PDCD2L expression indicated poor overall survival rate in ACC (P = 0.04), KICH (P = 0.0075), LAML (P = 0.0032), LGG (P = 0.013), LIHC (P = 0.044), MESO (P = 0.0077), and UVM(P = 0.016),while highPDCD2L expression revealed favorable overall survival rate in UCEC(P = 0.039) (Fig. 2A and B). Moreover, high PDCD2L expression also indicated unfavorable diseases free survival in ACC (P = 0.039), BLCA (P = 0.048), CESC (P = 0.014), KIRC (P = 0.031), KIRP (P = 0.041), LGG (P = 0.043), LIHC (P = 0.021), and UVM (P = 0.016). While high PDCD2L expression suggested favorable diseases free survival in PAAD (P = 0.043) (Fig. 2C and D). We also noticed that PDCD2L expression was not associated with the overall survival of BLCA, BRCA, CESC, CHOL, COAD, DLBC, ESCA, GBM, HNSC, KIRC, KIRP, LUAD, LUSC, OV, PAAD, PCPG, PRAD, READ, SARC, SKCM, STAD, TGCT, THCA, Thymoma (THYM) and UCS (data not shown). Furthermore, PDCD2L expression was not associated with the diseases free survival of BRCA, CHOL. COAD, DLBC, ESCA, GBM, HNSC, KICH, LAML, LUAD, LUSC, MESO, OV, PCPG, PRAD, READ, SARC, SKCM, STAD, TGCT, THCA, THYM, UCEC, and UCS (data not shown).

### Co-expression network of PDCD2L and enrichment pathway analysis

**Fig. 3:**
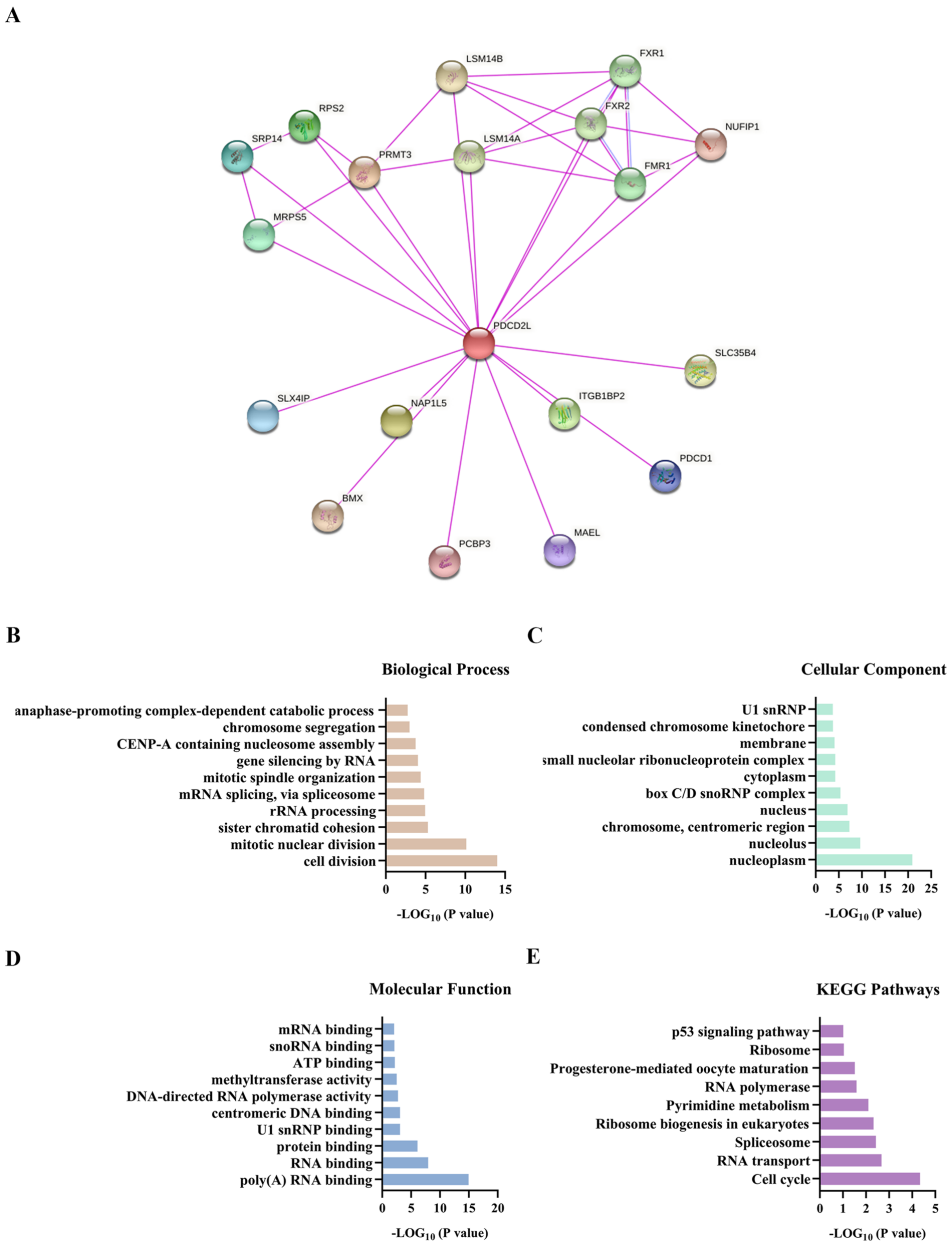
Co-expression network of PDCD2L and enrichment pathway analysis. **A** The binding proteins of PDCD2L by STRING website. **B** Biological process, **C** cellular component, **D** molecular function, **E** KEGG pathways based on PDCD2L binding proteins and interactive genes

Our above results suggested that PDCD2L might play an oncogene role in pan-cancer and could be used as a prognostic marker. However, the molecular mechanism of PDCD2L in tumorigenesis remains unknown. To explore the co-expression network and enrichment pathways of PDCD2L in pan-cancer, firstly, we predicted the binding proteins of PDCD2L by STRING website, which were validated by experiment. The result revealed that there were 18 proteins includingFXR2, LSM14B, LSM14A, FXR1, NUFIP1, SRP14, PRMT3, SLC35B4, SLX4IP, RPS2, BMX, FMR1, ITGB1BP2, MAEL, MRPS5, NAP1L5, PCBP3, and PDCD1binding to PDCD2L (Fig. 3A).Then we obtained the top 100 genes that closely related to PDCD2L from GEPIA2.By crossing two sets of data, we found that LSM14A was the only common gene in both groups. Further, GO and KEGG enrichment analysis were performed for the 117 gene. GO enrichment indicated that these genes were significantly related with poly(A) RNA binding, RNA binding, protein binding, U1 snRNP binding, centromeric DNA binding, DNA-directed RNA polymerase activity, methyltransferase activity, ATP binding, snoRNA binding, mRNA binding and others (Fig. 3B–D). Furthermore, we found that PDCD2Lwas involved in tumorigenesis through cell cycle, RNA transport, Spliceosome, Ribosome biogenesis in eukaryotes, Pyrimidine metabolism, RNA polymerase, Progesterone-mediated oocyte maturation, Ribosome, and p53 signaling pathway (Fig. 3E).

### Negative correlation between PDCD2L expression and CAF

**Fig. 4:**
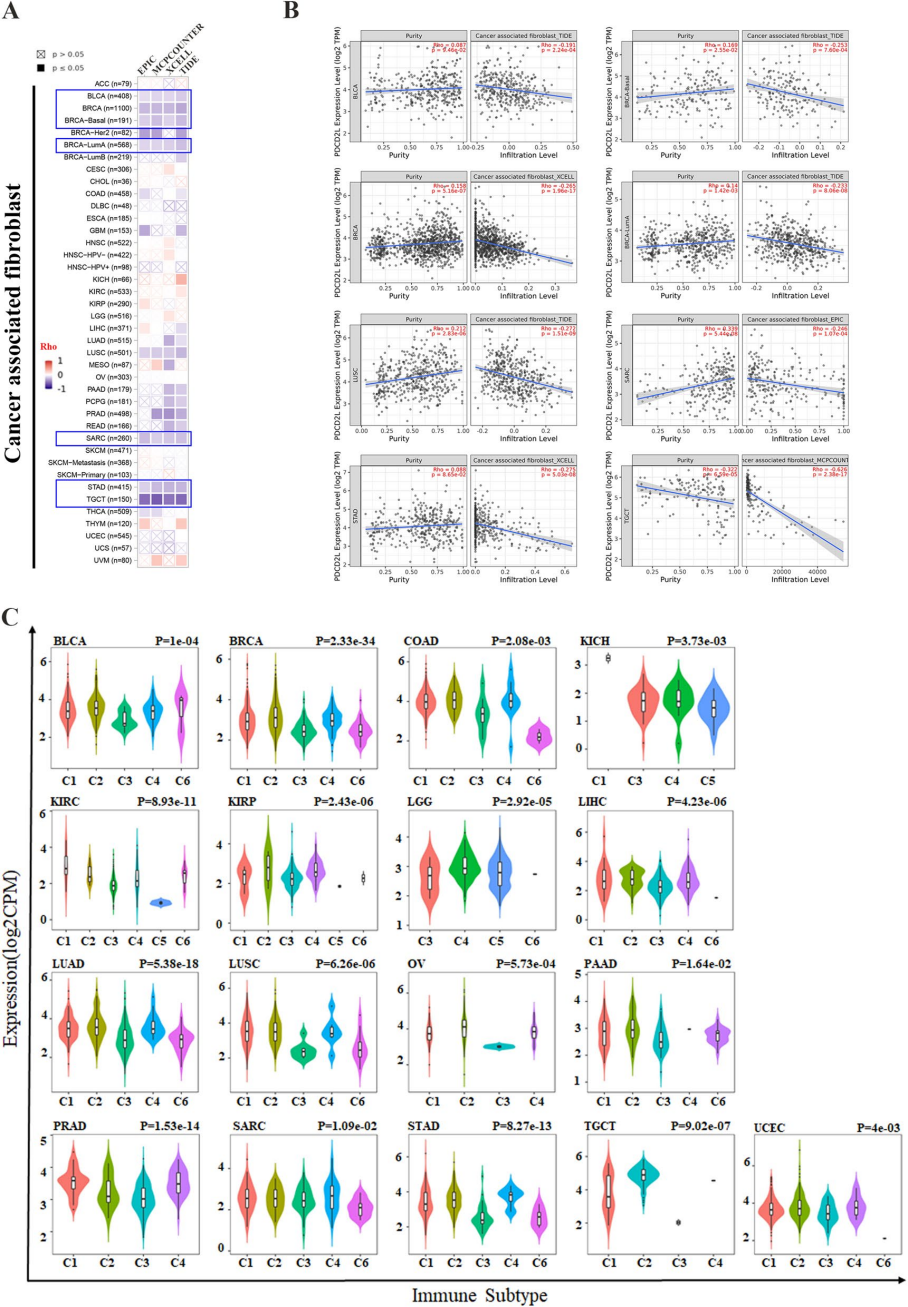
Negative correlation between PDCD2L expression and CAF. **A** and **B** correlation between PDCD2L expression and cancer associated fibroblasts was performed by TIMER2.0. **C** PDCD2L expression level in immune subtypes in BLCA, BRCA, COAD, KICH, KIRC, KIRP, LGG, LIHC, LUAD, LUSC, OV, PAAD, PRAD, SARC, STAD, TGCT and UCEC based on TISDB

The tumor microenvironment (TME) provides a crosstalk between tumor cells and the microenvironment and TME is composed of four major components: immune component, vascular component, extracellular matrix and stromal component. Immune component includes many immune cells such as T cells and B cells, while stromal component is composed of CAF and mesenchymal stem cells [18].Recently multiple studies have highlighted the vital role of CAF in cancer progression and immune response [19]. To elucidate the role of PDCD2L in TME, we analyzed the correlation between PDCD2L expression and TME components in pan-cancer by TIMER 2.0 website. We obtained a consistent result that PDCD2L expression was negatively related with CAF in BLCA, BRCA, BRCA-Basal, LUSC, SARC, STAD and TGCT by EPIC, MCPCOUNTER, XCELL and TIDE algorithms (Fig. 4A and B). To further investigate the underlying mechanism of the correlation between PDCD2L expression and CAF, we analyzed the expression markers of CAF in pan-cancer. We found that PDCD2L expression was significantly associated with immune subtype including C1(wound healing), C2(IFN-gamma dominant), C3(inflammatory), C4(lymphocyte depleted), C5(immunologically), C6(TGF-b dominant).

of BLCA, BRCA, COAD, KICH, KIRC, KIRP, LGG, LIHC, LUAD, LUSC, OV, PAAD, PRAD, SARC, STAD, TGCT and UCEC (Fig. 4C). While the expression of PDCD2L in the immune subtype of ACC, CESC, CHOL, ESCA, GBM, HNSC, MESO and PCPG was not statistically different (data not shown).

### Expression pattern of PDCD2L in single cell and its relationship with cancer functional status

**Fig. 5:**
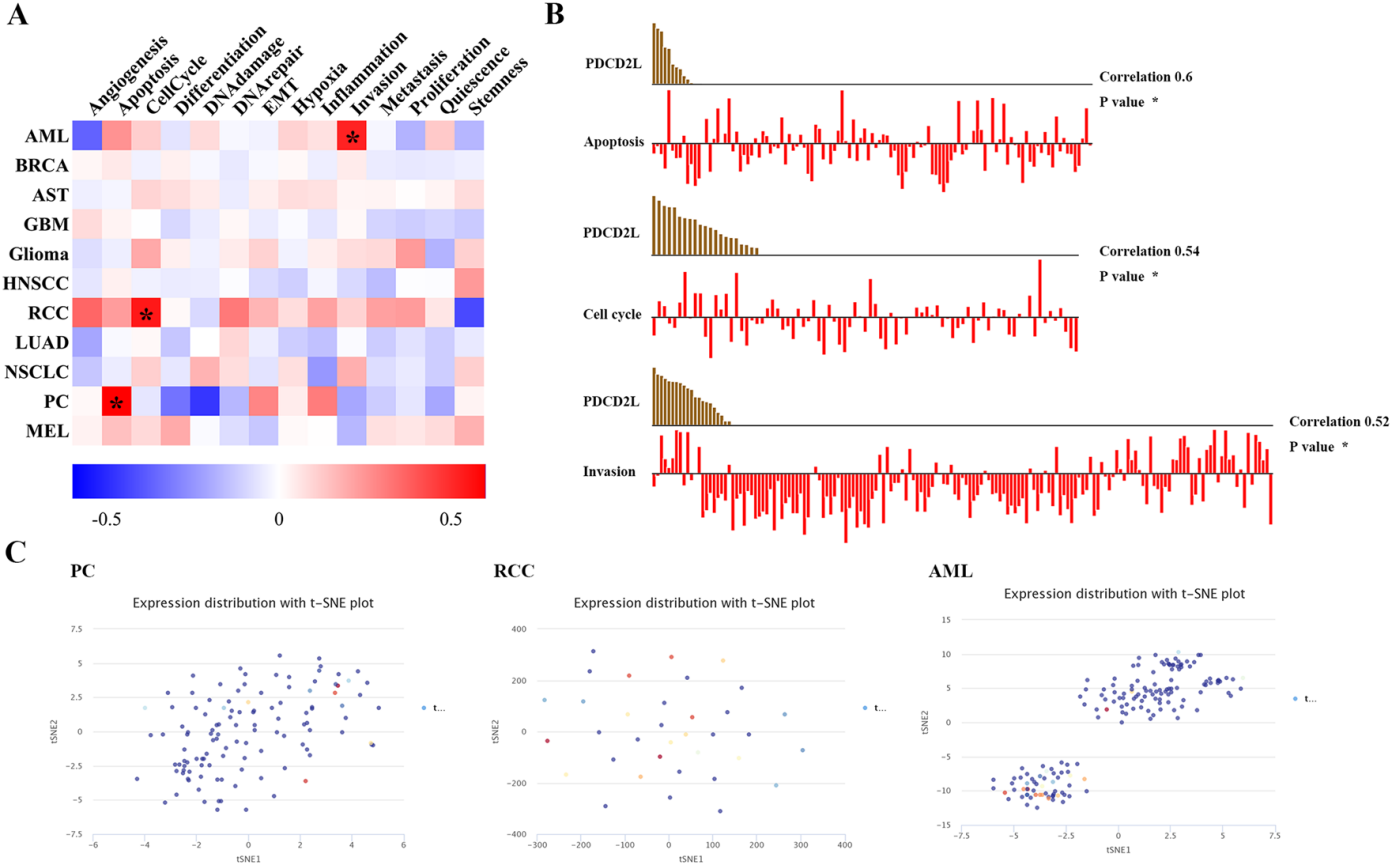
Expression pattern of PDCD2L in single cell sequencing and its correlation with tumor functional status. **A** Heatmap showed the correlation between PDCD2L expression and different tumor functional status based on CancerSEA database (*P < .05). **B** correlation between PDCD2L expression and three significantly different functional states based on CancerSEA database (*P < 0.05). **C** T-SNE diagram demonstrated PDCD2L expression profiles were in single cells of AML, PC and RCC samples, respectively

Since the complexity of tumor cells, single-cell transcriptomic sequencing is a key technique to analyze diverse cancer cells, immune cells, endothelial cells and stromal cells [20]. In order to verify the expression of PDCD2L in single cell in different cancers and its relationship with tumor functional status, we found that PDCD2L expression in prostatic cancer (PC) was significantly positively associated with apoptosis, PDCD2L expression in renal cell carcinoma (RCC) was significantly positively associated with cycle, and PDCD2L expression in Acute myeloid leukemia(AML) was significantly positively associated with invasion through CANCERSEA website (Fig. 5A). Figure 5B showed the relationship between PDCD2L expression and apoptosis in PC, PDCD2L expression and cell cycle in RCC, and PDCD2L expression and invasion in AML.PDCD2L expression profiles were shown in single cells of PC, RCC and AML by T-SNE diagram (Fig. 5C).The results suggest that PDCD2L might play a crucial role in the process of cancer progression.

### High PDCD2L expression in our CRC samples and down-regulation of PDCD2L expression promotes apoptosis in CRC cells

**Fig. 6:**
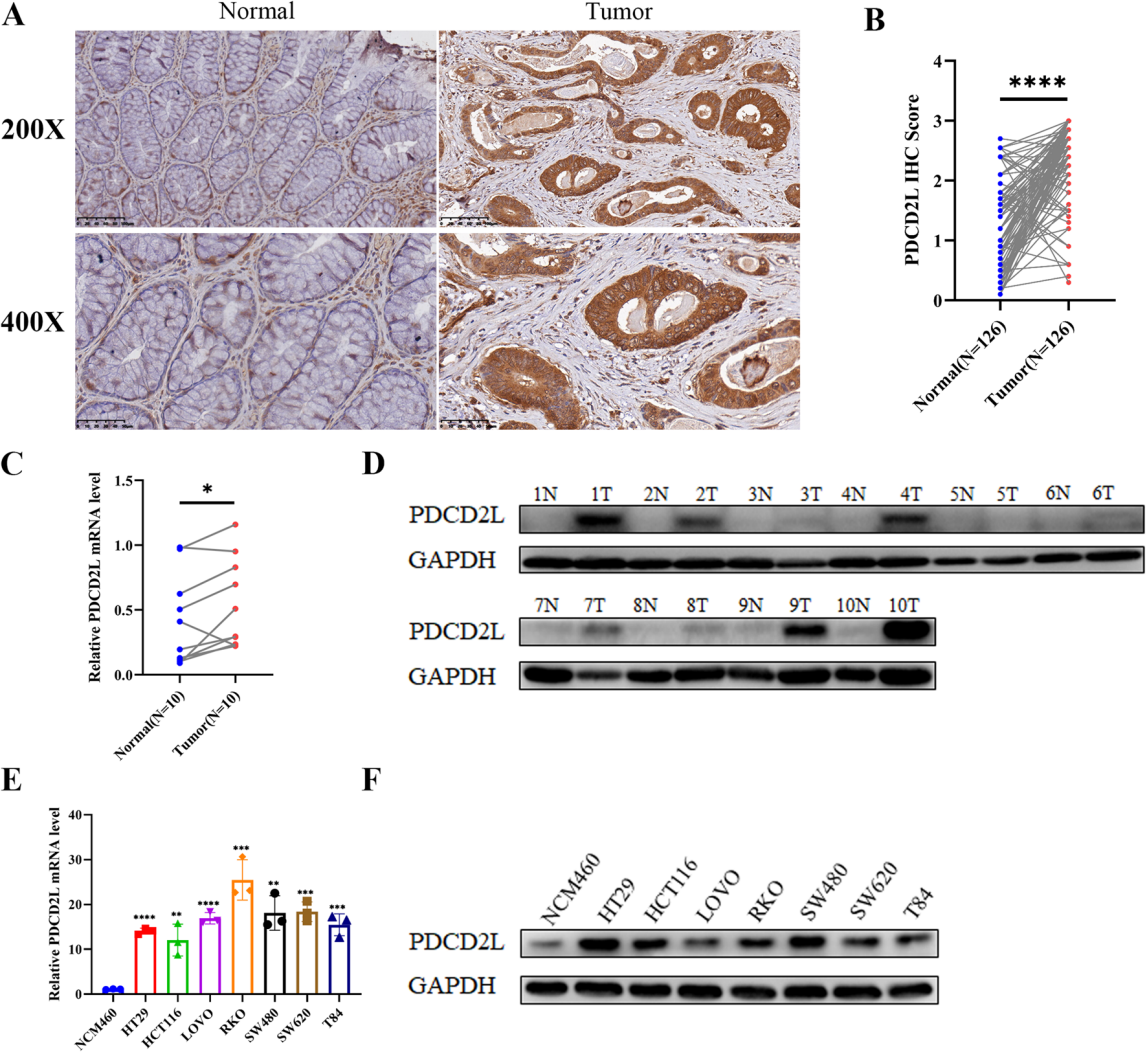
High PDCD2L expression in our CRC samples. **A** Positive PDCD2L signal was located in cytoplasm of carcinoma by IHC. **B** PDCD2L was highly expressed in our 126 pairs of CRC and corresponding non-tumor colorectal tissues by IHC. **C** and **D** transcription level and protein level of PDCD2L in fresh CRC tissues and adjacent non-tumor colorectal mucosa tissues. **E** and **F** transcription level and protein level of PDCD2L in CRC cell lines and NCM460. (** P < .01; *** P < .001; **** P < .0001)

According to the above results, PDCD2L was highly expressed in CRC tissues compared with non-tumor adjacent tissues. In order to validate PDCD2L expression in our CRC samples and study the role of PDCD2L in CRC progression. Firstly, we performed IHC to detect PDCD2L expression in 126 pairs of paraffin-embedded tissues of CRC and non-tumor adjacent colorectal mucosa tissues. Our result showed that positive PDCD2L signal was located in cytoplasm of carcinoma cell in our CRC samples (Fig. 6A). And PDCD2L was highly expressed in CRC tissues compared with non-tumor colorectal mucosa tissues (Fig. 6B). To analyze the correlation between PDCD2L expression and clinicopathological characteristics of CRC patients, we divided our samples were into two groups according to the median (score as 2.7) of PDCD2L expression, among which the low PDCD2L expression group was less than 2.7 and high PDCD2L expression group was above or equal 2.7.Also, our study showed that PDCD2L expression was significant associated with histological differentiation and sex of CRC patients, while PDCD2L expression was not differ in other characteristics such as age, tumor size, vascular tumor thrombus, perineural invasion, T classification, N classification, M classification and TNM stage (Table 1). However, PDCD2L expression was not associated with the overall survival and diseases free survival of CRC patients (data not shown). By collecting 10 pairs of fresh CRC tissues and corresponding adjacent tissues for qPCR and WB detection, we found that the transcription level and protein level of PDCD2L in CRC tissues were significantly higher than those in non-tumor adjacent colorectal mucosa tissues (Fig. 6C and D). We also detected PDCD2L mRNA and protein expression in CRC cell lines and colon mucosal epithelial cell line NCM460. The results showed that the transcription level and protein level of PDCD2L in CRC cell lines were significantly higher than those in NCM460 (Fig. 6E and F).

**Table 1:** PDCD2L expression and its association with clinicopathological features of CRC

	low-PDCD2L	high-PDCD2L	
Characteristics	No.(%)	No.(%)	P value
Age(years)			0.083
Mean ± SD	56.2 ± 14.1	59.4 ± 12.2	
< 60	45(60.8)	39(47.0)	
≥ 60	29(39.2)	44(53.0)	
Sex			0.002
Male	56(75.7)	43(51.8)	
Female	18(24.3)	40(48.2)	
Tumor size(cm)			0.519
Mean ± SD	4.9 ± 2.4	4.2 ± 1.5	
< 5	42(58.3)	52(63.4)	
≥ 5	30(41.7)	30(36.6)	
Histological differentiation			0.041
Poorly	18(24.7)	10(12.0)	
Moderately and well	55(75.3)	73(88.0)	
T classification			0.332
T1 + T2	10(13.5)	16(19.3)	
T3 + T4	64(86.5)	67(80.7)	
N classification			0.585
N0	36(48.6)	44(53.0)	
N1 + N2	38(51.4)	39(47.0)	
M classification			0.851
M0	69(93.2)	78(94.0)	
M1	5(6.8)	5(6.0)	
TNM stage			0.463
I + II	34(45.9)	43(51.8)	
III + IV	40(54.1)	40(48.2)	
Vascular tumor thrombus			0.56
Absent	64(86.5)	69(83.1)	
Present	10(13.5)	14(16.9)	
Perineural invasion			0.734
Absent	57(77.0)	62(74.7)	
Present	17(23.0)	21(25.3)	

**Fig. 7:**
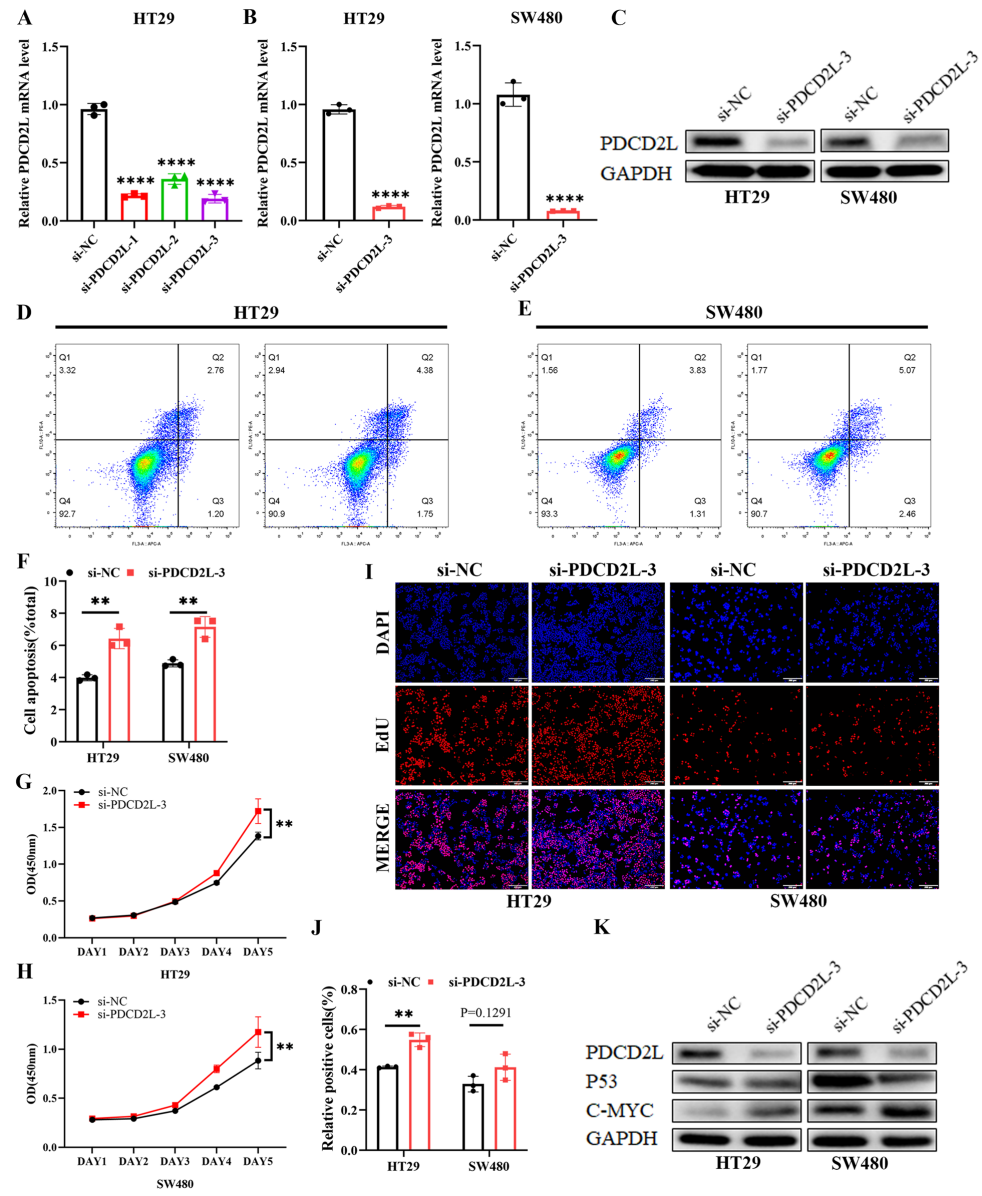
Down-regulation of PDCD2L expression promotes apoptosis in CRC cells. **A** PDCD2L expression was downregulated in HT29cells transfected with 3 different PDCD2L siRNA sequences by qPCR. **B** and **C** transcription level and protein level of PDCD2L in HT29 and SW480 were significantly downregulated by si-PDCD2L-3 transfection, respectively. **D**–**F** PDCD2L knockdown significantly increased the total apoptosis rate of HT29 and SW480. **G**–**J** cell proliferation was assessed by CCK assay and EdU assay. **K** PDCD2L-related signaling pathways were determined by western blot. (** P < .01; **** P < .0001)

Next, we performed functional experiment in HT29and SW480 cell lines, which had high expression of PDCD2L.PDCD2L mRNA expression decreased in HT29 transfected with three different PDCD2L siRNA by qPCR, respectively. The effect of si-PDCD2L-3 was the most significant (Fig. 7A). Therefore, we selected si-PDCD2L-3for functional experiments. The transcription level and protein level of PDCD2L in HT29 and SW480 transfected with si-PDCD2L-3decreased significantly by (Fig. 7B and C). While the above single cell sequencing results suggested that PDCD2L expression was closely related with tumor apoptosis, we focused on the effect of PDCD2L on apoptosis of CRC cells. Apoptosis assay showed that PDCD2L expression knockdown significantly increased the total apoptosis rate of HT29 and SW480, respectively (Fig. 7D–F). To investigate the effect of PDCD2L on celluar proliferation of CRC cells, we performed CCK8 and EdU assays using siRNA. Interestingly, CCK8 assay showed that that PDCD2L knockdown significantly enhanced the proliferation of HT29 and SW480 compared with the control groups (Fig. 7G and H), which were verified by EdU assay (Fig. 7I and J). Our result revealed that downregulation of PDCD2L enhanced total apoptosis rate and proliferation of CRC cells, we hypothesized that PDCD2L might be involved in apoptosis induced proliferation (AiP) on the progression of CRC. Since KEGG results suggested that PDCD2L was significantly associated with P53 signaling pathways, our result revealed that PDCD2L knockdown inhibited P53 expression and increased c-MYC expression by western blot assay (Fig. 7K).

## Discussion

Pan-cancer analysis provides a comprehensive understanding on the molecular aberrations of various cancers and is helpful for finding biomarkers in early diagnosis and target therapies of cancers. Leiserson et al. have identified 14 remarkable mutated subnet works by investigating pan-cancer data of The Cancer Genome Atlas (TCGA), which includes many noted cancer signaling pathways [21]. Aran et al. systematically analyzed the purity of pan-cancer based on the Cancer Genome Atlas, then identified a novel immunotherapy gene signature for many cancers [22]. Ma et al. provided new insight into pan-cancer analysis of 1,699 paediatric cancers, which present a general genomic landscape for paediatric leukaemias and solid tumors [23]. Since cancer-associated DNA methylation may affect gene expression, Saghafinia et al. analyzed the changes of DNA methylation in pediatric tumors, which provided a pan-cancer architecture of aberrant DNA methylation [24].Priestley et al. performed a pan-cancer analysis of metastatic solid tumor genomes, and found that the difference of mutational map and driver genes between metastatic tumor genomes and primary tumors genomes is not obvious [25]. These studies have showed the correlation between tumorigenesis and genome, transcriptome and epigenomics in pan-cancer. The expression of PDCD2L did not significantly affect apoptosis in HEK293T cells by different treatments [2]. Augmented levels of PDCD2L protein inhibit proliferation of HEK293T cells and block cell cycle progression at S phase [3]. PDCD2L overexpression in pancreatic beta-cell enhanced palmitate-induced apoptotic rate [4]. PDCD2L is evolutionarily conserved in development [5].Since the role of PDCD2L in cancer progression remains unclear, we systematically analyzed the expression and prognosis value of PDCD2L in pan-cancer. Our study revealed that PDCD2L was highly expressed in various cancers compared with the corresponding non-tumor tissues. The result suggests PDCD2L might be as oncogene in these cancers.

Several studies of pan-cancer analysis have addressed the significance of aberrant expressed genes in the development or progression of CRC. To evaluate the role of antiapoptotic BCL-2 proteins on preclinical CRC models, Scherr et al. have found that BCL-XL is overactivated in CRC, which provides a promising therapeutic target for CRC by analyzing a pan-cancer cohort, [26].High ANKRD6 expression is correlated with poor prognosis of colon cancer based on a pan-cancer analysis, which suggests ANKRD6 plays an important role in the progression of colon cancer [27]. According to a pan-cancer analysis, RRM2ishighlyexpressed in many cancers, Further experiment has found that RRM2 is significantly upregulated in CRC.RRM2 might serve as a novel potential target for CRC therapy [28]. Furthermore, we also detected PDCD2L expression in our CRC samples and found that the expression of PDCD2L is significantly higher in CRC tissues than that in corresponding non-tumor colorectal mucosa tissues. The relationship between the expression of PDCD2L and the prognosis of patients with CRC needs further study by expanding sample size.

Currently, tumor microenvironment (TME) has been a hot and trending spot in tumor research [18]. As a major component of TME, CAF has multiple pro-tumorigenic functions during tumorigenesis. Francescone et al. have found that Netrin G1promotes PDAC progression by increasing the tumor-promoting role of CAFs [29]. CAF promotes cholangiocarcinoma progression through a mice model. Pan-CAF signature is significantly correlated with poor survival in patients with cholangiocarcinoma and tumor recurrence [30]. Recent study has revealed that pancreatic cancer cells co-culture with CAF can promote cancer stemness [31]. Intriguingly, some studies showed that CAF could inhibit tumor growth and invasion. Ozdemir et al. have reported that depletion of CAF can augment pancreas cancer growth, which suggests that CAF may play a anti-tumor role in some tumors [32].Our above study showed that PDCD2L expression was negatively correlated with CAF in many cancers including BRCA, BRCA-Basal, LUSC, SARC, STAD and TGCT. PDCD2L might play an oncogene role in various cancers, the underlining mechanism of PDCD2L regulating CAF needs further study. Recently six immune subtypes: C1 (Wound Healing), C2 (IFN-γ Dominant), C3 (Inflammatory), C4 (Lymphocyte Depleted), C5 (Immunologically Quiet), and C6 (TGF-β Dominant) were first identified by an extensive immunogenomic analysis of 33 different types of cancer. Among them, C1 was characterized by increased angiogenesis gene expression, high proliferation rate and Th2 cells tend to adaptive immune infiltration. While the most apparent feature of C2 was the highest M1/M2 macrophage polarization and the optimal TCR diversity, and C2 also exhibited a boost proliferation rate and an augment CD8 signal. C3 showed an up-regulation of Th17 and Th1 genes, low or moderate cancer cell proliferation, and the aneuploidy and overall somatic copy number alterations was also decreased. C4 was highlighted by macrophage characteristics and higher M2 response, while Th1 was inhibited. C5 exhibited the lowest lymphocyte and the strongest macrophage response, which mainly conducted by M2 macrophages. C6 subtype showed the highest TGF-β characteristics and high lymphocyte infiltration, in which type I and type II T cells were evenly distributed [33]. The novel immune subtype can distinct multiple heterogeneous tumors, which may benefit the targeted immune therapy of cancer patients. In our study, PDCD2L expression is significant aberrant expressed in different immune subtypes of BLCA, BRCA, COAD, KICH, KIRC, KIRP, LGG, LIHC, LUAD, LUSC, OV, PAAD, PRAD, SARC, STAD, TGCT and UCEC, which suggested that PDCD2L might play an important role in cancer immune therapy.

Since single-cell transcriptomic sequencing study suggested that PDCD2L expression was significantly associated with apoptosis in PC. To elucidate the role of PDCD2L in CRC progression, apoptosis rate of CRC cells significantly increased by downregulating PDCD2L expression in HT29 and SW480, respectively. In addition, PDCD2L knockdown also enhanced the proliferation of CRC cells. The results suggest that PDCD2L might inhibit the apoptosis and proliferation of CRC cells. Apoptosis-induced proliferation (AiP), a form characterized by a compensatory proliferation mechanism, which is trigger by apoptosis [34]. Aip is critical for tissue development and regeneration, and persistent Aip may cause tumor repopulation and drives angiogenesis following therapy [35]. Recent study shows that inhibiting DDX3X expression induced cell apoptosis, and also loss of DDX3X caused compensatory proliferation [36]. Moreover, downregulation of TNFR2, but not TNFR1, triggers apoptosis of Schwann cells, and the apoptosis also increases Schwann cell proliferation [37]. Our study first suggests that PDCD2L knockdown might trigger apoptosis induced proliferation in CRC cells. However, the mechanism of PDCD2L regulating Aip in CRC needs further investigation.

In conclusion, our study first provides a comprehensive analysis of PDCD2L in pan-cancer and found that PDCD2L expression was high in various cancers and negatively associated with CAF. Our validation experiment found that PDCD2L expression was significantly higher in CRC samples than that in corresponding non-tumor tissues, which suggests that PDCD2L could be served as a biomarker of CRC. PDCD2L might play an important role in CRC progression by participating in apoptosis induced proliferation of CRC cells.

## Supplementary Information

**Additional file 1: Figure S1**. The association between PDCD2L expression and molecular subtype of COAD, READ, and some cancers.

## Data Availability

The original contributions presented in the study are included in the article/supplementary material. Further inquiries can be directed to the corresponding authors.

## References

[1] Sung H, Ferlay J, Siegel RL (2021). Global cancer statistics 2020: GLOBOCAN estimates of incidence and mortality worldwide for 36 cancers in 185 countries. CA Cancer J Clin.

[2] Chen Q, Qian K, Yan C (2005). Cloning of cDNAs with PDCD2(C) domain and their expressions during apoptosis of HEK293T cells. Mol Cell Biochem.

[3] Chen Q, Yan C, Yan Q (2008). The novel MGC13096 protein is correlated with proliferation. Cell Biochem Funct.

[4] Yin Y, Yong W, Yu J (2016). Pdcd2l Promotes palmitate-induced pancreatic beta-cell apoptosis as a FoxO1 target gene. PLoS ONE.

[5] Houston BJ, Oud MS, Aguirre DM (2020). Programmed cell death 2-like (Pdcd2l) is required for mouse embryonic development. G3 (Bethesda).

[6] Rhodes DR, Yu J, Shanker K (2004). ONCOMINE: a cancer microarray database and integrated data-mining platform. Neoplasia.

[7] Rhodes DR, Kalyana-Sundaram S, Mahavisno V (2007). Oncomine 3.0: genes, pathways, and networks in a collection of 18,000 cancer gene expression profiles. Neoplasia.

[8] Li T, Fu J, Zeng Z (2020). TIMER2.0 for analysis of tumor-infiltrating immune cells. Nucleic Acids Res.

[9] Li B, Severson E, Pignon JC (2016). Comprehensive analyses of tumor immunity: implications for cancer immunotherapy. Genome Biol.

[10] Li T, Fan J, Wang B (2017). TIMER: a web server for comprehensive analysis of tumor-infiltrating immune cells. Cancer Res.

[11] Tang Z, Kang B, Li C (2019). GEPIA2: an enhanced web server for large-scale expression profiling and interactive analysis. Nucleic Acids Res.

[12] Ru B, Wong CN, Tong Y (2019). TISIDB: an integrated repository portal for tumor-immune system interactions. Bioinformatics.

[13] Szklarczyk D, Gable AL, Nastou KC (2021). The STRING database in 2021: customizable protein-protein networks, and functional characterization of user-uploaded gene/measurement sets. Nucleic Acids Res.

[14] da Huang W, Sherman BT, Lempicki RA (2009). Bioinformatics enrichment tools: paths toward the comprehensive functional analysis of large gene lists. Nucleic Acids Res.

[15] da Huang W, Sherman BT, Lempicki RA (2009). Systematic and integrative analysis of large gene lists using DAVID bioinformatics resources. Nat Protoc.

[16] Yuan H, Yan M, Zhang G (2019). CancerSEA: a cancer single-cell state atlas. Nucleic Acids Res.

[17] Yang Y, Ma Y, Gao H (2021). A novel HDGF-ALCAM axis promotes the metastasis of Ewing sarcoma via regulating the GTPases signaling pathway. Oncogene.

[18] Mhaidly R, Mechta-Grigoriou F (2021). Role of cancer-associated fibroblast subpopulations in immune infiltration, as a new means of treatment in cancer. Immunol Rev.

[19] Gentric G, Mechta-Grigoriou F (2021). Tumor cells and cancer-associated fibroblasts: an updated metabolic perspective. Cancers (Basel).

[20] Li Y, Jin J, Bai F (2021). Cancer biology deciphered by single-cell transcriptomic sequencing. Protein Cell.

[21] Leiserson MD, Vandin F, Wu HT (2015). Pan-cancer network analysis identifies combinations of rare somatic mutations across pathways and protein complexes. Nat Genet.

[22] Aran D, Sirota M, Butte AJ (2015). Systematic pan-cancer analysis of tumour purity. Nat Commun.

[23] Ma X, Liu Y, Liu Y (2018). Pan-cancer genome and transcriptome analyses of 1,699 paediatric leukaemias and solid tumours. Nature.

[24] Saghafinia S, Mina M, Riggi N (2018). Pan-cancer landscape of aberrant DNA methylation across human tumors. Cell Rep.

[25] Priestley P, Baber J, Lolkema MP (2019). Pan-cancer whole-genome analyses of metastatic solid tumours. Nature.

[26] Scherr AL, Mock A, Gdynia G (2020). Identification of BCL-XL as highly active survival factor and promising therapeutic target in colorectal cancer. Cell Death Dis.

[27] Bai R, Wu D, Shi Z (2021). Pan-cancer analyses demonstrate that ANKRD6 is associated with a poor prognosis and correlates with M2 macrophage infiltration in colon cancer. Chin J Cancer Res.

[28] Liu Q, Guo L, Qi H (2021). A MYBL2 complex for RRM2 transactivation and the synthetic effect of MYBL2 knockdown with WEE1 inhibition against colorectal cancer. Cell Death Dis.

[29] Francescone R, Barbosa Vendramini-Costa D, Franco-Barraza J (2021). Netrin G1 promotes pancreatic tumorigenesis through cancer-associated fibroblast-driven nutritional support and immunosuppression. Cancer Discov.

[30] Affo S, Nair A, Brundu F (2021). Promotion of cholangiocarcinoma growth by diverse cancer-associated fibroblast subpopulations. Cancer Cell.

[31] Nallasamy P, Nimmakayala RK, Karmakar S (2021). Pancreatic tumor microenvironment factor promotes cancer stemness via SPP1-CD44 axis. Gastroenterology.

[32] Ozdemir BC, Pentcheva-Hoang T, Carstens JL (2014). Depletion of carcinoma-associated fibroblasts and fibrosis induces immunosuppression and accelerates pancreas cancer with reduced survival. Cancer Cell.

[33] Thorsson V, Gibbs DL, Brown SD (2018). The immune landscape of cancer. Immunity.

[34] Fogarty CE, Diwanji N, Lindblad JL (2016). Extracellular reactive oxygen species drive apoptosis-induced proliferation via drosophila macrophages. Curr Biol.

[35] Fogarty CE, Bergmann A (2017). Killers creating new life: caspases drive apoptosis-induced proliferation in tissue repair and disease. Cell Death Differ.

[36] Chan CH, Chen CM, Lee YW (2019). DNA damage, liver injury, and tumorigenesis: consequences of DDX3X loss. Mol Cancer Res.

[37] Gao Z, Min C, Xie H (2020). TNFR2 knockdown triggers apoptosis-induced proliferation in primarily cultured Schwann cells. Neurosci Res.

