# Supplementary figures and images for: Pan-cancer analysis of oncogenic role of Programmed Cell Death 2 Like (PDCD2L) and validation in colorectal cancer
Source: Cancer Cell Int. 2022 Feb 25;22:100. doi: 10.1186/s12935-022-02525-x (PMC8881831; doi:10.1186/s12935-022-02525-x)

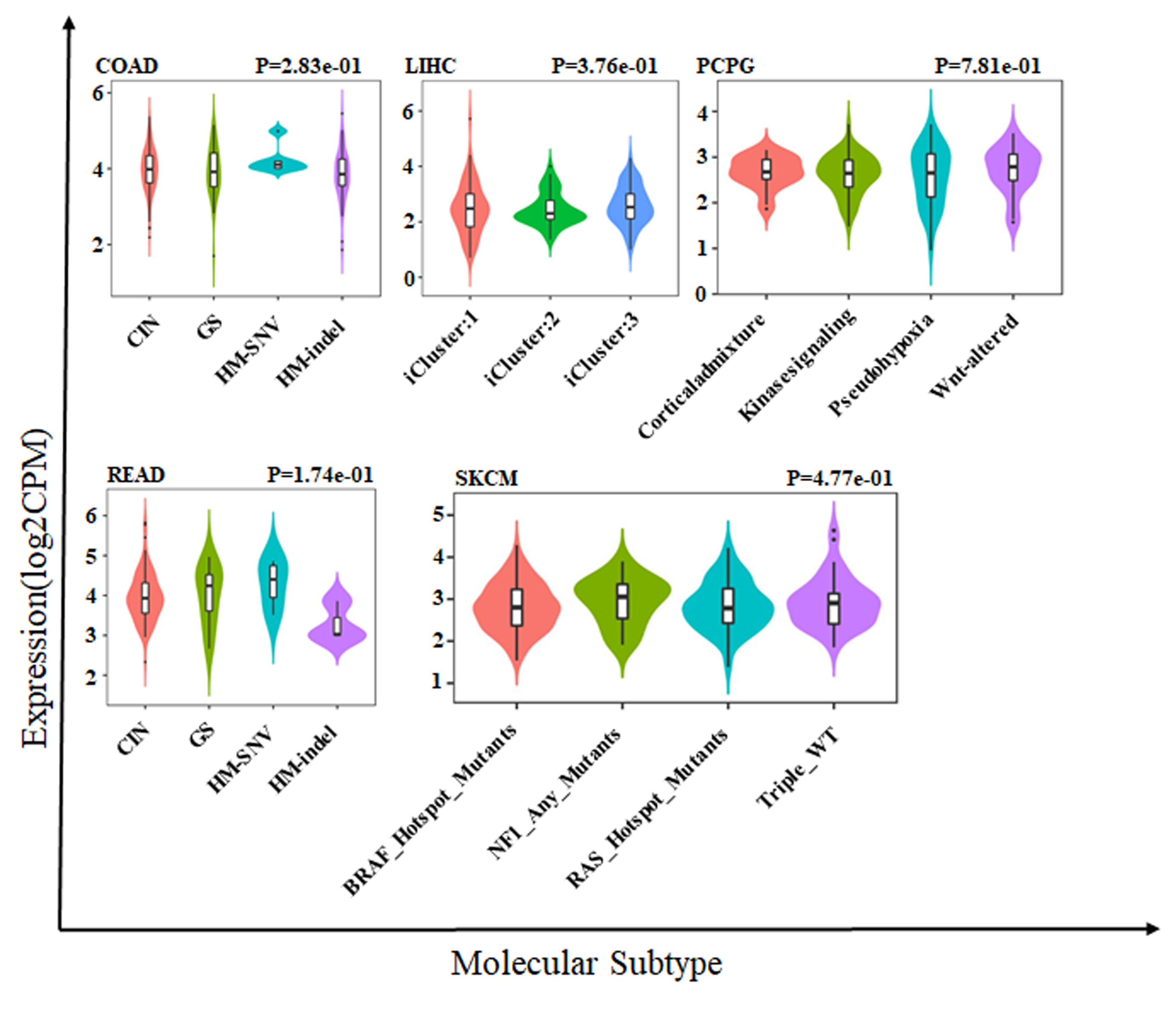

Supplement: Supplementary file 1 — Additional file 1: Figure S1. The association between PDCD2L expression and molecular subtype of COAD, READ, and some cancers. [file 12935_2022_2525_MOESM1_ESM.tif]
